# Metabolomic profiling of blood from Nellore and Angus cattle under heat stress

**DOI:** 10.1007/s44154-026-00303-7

**Published:** 2026-04-21

**Authors:** Gabriele Rocchetti, Michela Errico, Emanuele Capra, Marco Milanesi, Giulia Fappani, Jose Fernando Garcia, Guilherme de Paula Nogueira, Yuri Tani Utsunomiya, Luigi Lucini, Ana Maria Perez OBrien, Paolo Ajmone Marsan

**Affiliations:** 1 Department of Animal Science, Food and Nutrition, Università Cattolica del Sacro Cuore, Via Emilia Parmense 84, Piacenza, 29122 Italy; 2 Institute of Agricultural Biology and Biotechnology (IBBA), National Research Council (CNR), Lodi, 26900 Italy; 3 Department for Innovation in Biological, Agri-Food, and Forestry Systems - DIBAF, University of Tuscia, Viterbo, 01100 Italy; 4 Department of Production and Animal Health, School of Veterinary Medicine, Araçatuba, São Paulo State University (UNESP), Araçatuba, Brazil; 5 Department for Sustainable Food Process (DiSTAS), Università Cattolica del Sacro Cuore, Via Emilia Parmense 84, Piacenza, 29122 Italy; 6 Agropartners Consulting, Rua Floriano Peixoto 120, Araçatuba, 1601020 Brazil; 7 CREI Research Center, Università Cattolica del Sacro Cuore, Via Emilia Parmense 84, Piacenza, 29122 Italy

**Keywords:** Thermotolerance, Integrative omics, Purines metabolism, Cattle, DNA methylation, Biomarkers, Phospholipids

## Abstract

Heat stress (HS) negatively affects cattle welfare, productivity, and sustainability, with marked differences between heat-tolerant breeds such as Nellore and heat-susceptible breeds such as Angus. In this study, an untargeted UHPLC-Orbitrap-MS metabolomic approach was used to investigate breed-specific differences in the blood metabolome associated with HS. Blood samples were collected from the same Nellore and Angus steers during peak HS exposure and after recovery, allowing both inter-breed and within-animal longitudinal comparisons. Multivariate analyses revealed a clear metabolic divergence between breeds during the challenge period, involving metabolites related to amino acid metabolism, lipid remodeling, energy pathways, and steroid metabolism. Longitudinal analyses accounting for repeated measures showed pronounced breed-specific metabolic responses. Nellore cattle displayed extensive and statistically robust metabolic changes across phases, with 114 metabolites remaining significant after false discovery rate (FDR) correction, including amino acids and peptides, nucleotide derivatives, carbohydrates, acyl-carnitines, steroids, and tetrapyrrole-related compounds. In contrast, Angus cattle showed only a limited number of FDR-significant metabolites, indicating a less consistent systemic response to HS. Pathway analysis identified porphyrin, purine, and pyrimidine metabolism as key pathways modulated by HS, particularly in Nellore cattle. Overall, these results suggest that heat tolerance is associated with coordinated metabolic adaptations rather than isolated metabolite changes. Further studies on larger cohorts are needed to validate the functional relevance of these findings.

**Supplementary Information:**

The online version contains supplementary material available at 10.1007/s44154-026-00303-7.

## Introduction

Heat stress (HS) is a significant environmental challenge affecting livestock production worldwide, particularly in regions with high temperatures and humidity (Giannone et al. 2023). The impact of HS on cattle includes reduced feed intake, impaired growth and reproduction, and increased susceptibility to diseases, ultimately leading to substantial economic losses for the livestock industry (Chen et al. 2024; Khan et al. 2023). Therefore, understanding the physiological and metabolic responses to HS is crucial to developing strategies to enhance the tolerance of cattle breeds.

Among cattle breeds, Nellore (*Bos taurus indicus*) and Angus (*Bos taurus*) (Del Corvo et al. 2021; Utsunomiya et al. 2019) show contrasting responses to HS. These breeds belong to different subspecies that originated from two independent domestication events: *B. taurus indicus* (zebu cattle) was domesticated in the Indian subcontinent, while *B. taurus taurus* (taurine cattle) was domesticated in the Near East. Nellore cattle, originating from tropical climates, are known for their superior heat tolerance compared to the Angus breed, which is primarily adapted to temperate regions (Santos et al. 2023). Despite their inherent heat tolerance, direct solar radiation can still compromise Nellore cattle's thermal balance when raised in pasture systems (Santos et al. 2023). The genetic and physiological mechanisms underlying differences in the heat response of these breeds have been the subject of extensive research (Meneses et al. 2021; 2013). However, the metabolic pathways associated with HS tolerance in Nellore cattle and susceptibility in Angus cattle remain poorly understood.

In our previous study (Del Corvo et al. 2021), we investigated deoxyribonucleic acid (DNA) methylation profiles in blood samples of Nellore and Angus steers exposed to the sun during the high temperature season of the summer in the Northwestern region of the Sao Paulo state (Brazil). Interestingly, DNA methylation analysis identified breed-specific patterns with different methylation responses to stress and post HS period. This stimulated us to investigate further the biological significance of some of the changes seen as a function of HS. The comprehensive analysis of metabolites within a biological system with no a priori hypothesis, termed untargeted metabolomics, is a powerful approach to elucidate the biochemical responses to environmental stressors such as HS (Feng et al. 2022; Yue et al. 2020). Untargeted metabolomics can identify and eventually quantify a wide array of metabolites, potentially providing insights into the metabolic adjustments that occur in response to HS. Blood is one of the most readily collected biological fluids and has been widely studied using targeted and untargeted metabolomic approaches (González-Domínguez et al. 2020). Therefore, comparing blood metabolomes of HS tolerant and susceptible breeds may uncover specific metabolites and metabolic pathways associated with heat tolerance.

In this study, we used untargeted metabolomics, based on ultra-high-performance liquid chromatography-high resolution mass spectrometry (UHPLC-HRMS), to investigate the differences in blood metabolomes between HS tolerant Nellore and HS susceptible Angus cattle. Our goal was to identify key metabolites and metabolic pathways that are affected by HS and contribute to the tolerance of Nellore cattle to HS and understand the metabolic disruptions experienced by Angus cattle under similar conditions. These metabolic differences could be the foundation for developing targeted interventions to enhance HS tolerance in cattle. The findings obtained have the potential to inform management practices aimed at improving heat tolerance in cattle, thereby enhancing animal welfare and production efficiency in HS environments. Additionally, the identified blood metabolites may serve as biomarkers to select heat-tolerant animals in breeding programs, contributing to the sustainability of livestock production and adaptation to climate change.

## Results

### Unsupervised and supervised statistics on metabolomic profiles to evaluate HS condition

The untargeted metabolomics facilitated the putative annotation of 1412 blood metabolites (representing the global detectable metabolome, not differentially expressed features), with an annotation confidence level 2 according to the spectral information reported on the comprehensive Bovine Metabolome Database (BDMB). All the metabolites identified are reported in the additional file (sheet No. 1), with their relative abundance values, MS1 and MSMS spectral information, and additional annotation-based information (including reference *m/z*, total identification scores, InChIKey specifications, and others). Among the identified metabolites, N-acetylcadaverine, LysoPE(24:1(15Z)/0:0), and adipate semialdehyde showed the lowest RSD% values, being 3.77, 3.87, and 4.47, respectively. An over-representation plot was then used to group the all the metabolites identified in the most abundant chemical classes; as a general consideration, amino acids, peptides and analogues were the most represented chemical classes (accounting for a total of 281 compounds), followed by carbohydrates and conjugates (total hits: 90), and fatty acids and conjugates (total hits: 63).

**Fig. 1:**
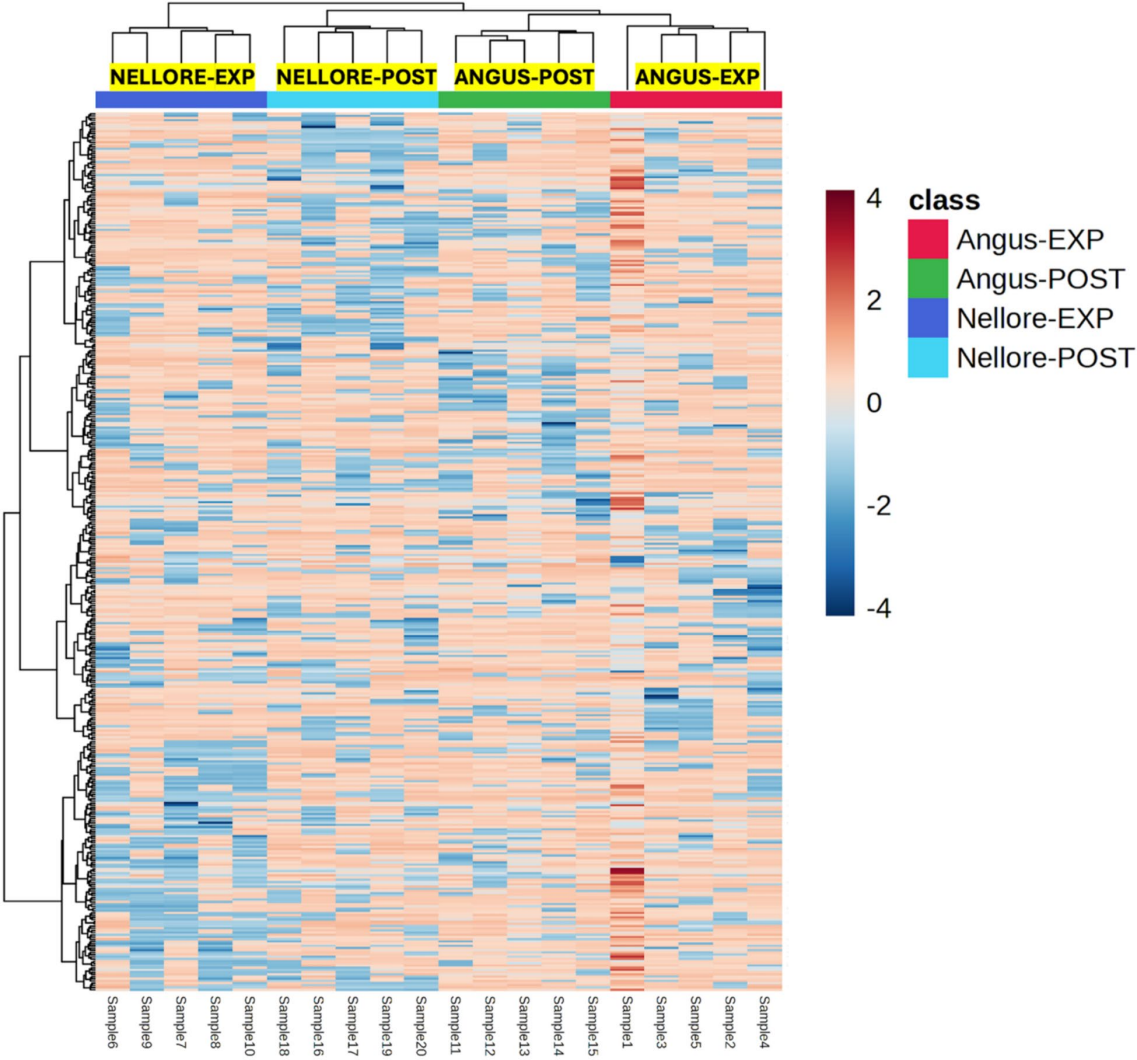
Heat map resulting from unsupervised hierarchical cluster analysis (HCA) on the 400 most significant blood metabolites discriminating Angus-EXP, Angus-POST, Nellore-EXP and Nellore-POST identified by the PLS-DA

The unsupervised hierarchical clustering approach (HCA) on all the annotated blood metabolites did not provide distinct patterns across treatments, as given by both the heat map and dendrogram (additional file; sheet No. 2). This was likely due to high biological variability and the prevalence of blood metabolites showing insignificant differences between breeds. Therefore, we carried out an averaged HCA analysis considering the 400 metabolites separating the four groups Angus-EXP, Angus-POST, Nellore-EXP, Nellore-POST in a partial least square discriminant analysis (PLS-DA), using the software MetaboAnalyst 6.0. A first cluster was identified, separating Nellore-EXP from the other blood samples (Fig. 1). The second subcluster was seen in Nellore-POST, together with Angus-POST and Angus-EXP blood samples.

OPLS-DA supervised modelling was then used as a supervised approach to maximize the covariance between the different sample replicates and optimize sample group separation between Angus-EXP, Angus-POST, Nellore-EXP and Nellore-POST. The orthogonal latent vector identified the breed effect, with the HS response as the secondary effect. The OPLS-DA model goodness of fit and prediction parameters were adequate, with the cumulative R^2^(cum) = 0.992 and the Q^2^(cum) = 0.883, and are reported as additional file (sheet No. 3).

### Blood metabolites as a function of breed during the challenge period

A supervised OPLS-DA model was used to reveal the specific metabolomic response of Angus and Nellore cattle during the challenge period (EXP). The OPLS-DA score plot (additional file; sheet No. 4) discriminated the metabolomic profile of the samples from the two breeds, showing a very high prediction ability (Q^2^ = 0.917). A total of 20 blood metabolites (Table 1) showed a very significant prediction ability (VIP score > 1.4; AUC value = 1; adjusted *p*-values < 0.05), including amino acids and derivatives, lipids and derivatives, organic acids, small peptides, circulating metabolites from the diet (such as phenolics and terpenoids), steroids and hormonal metabolites. The highest VIP score (3.17) and prediction ability (as a function of breed) was for the steroid 4-Hydroxyestrone, which had an increase accumulation in Angus-EXP blood samples *vs* Nellore (Log_2_FC = 3.79). The other most discriminating VIP metabolites for the pairwise comparison of Angus *vs.* Nellore during the challenge period (EXP) are reported in Table 1. This latter derive exclusively from inter-breed comparisons within the EXP phase and therefore do not involve repeated measures.

**Table 1:** Discriminant VIP compounds (from supervised OPLS-DA) in the pairwise comparison of Angus (A) *vs*. Nellore (N) blood metabolomes during the challenge period (EXP), owning a VIP score > 1.4 and a ROC AUC = 1. Each compound is reported with its VIP score, Log_2_ Fold-Change (FC), and significance (ANOVA, *p* < 0.05, with false discovery rate correction)

VIP metabolite (A *vs* N)	Chemical classification	VIP score	Log_2_FC (A *vs* N)	Significance *(p*-value)
4-Hydroxyestrone	Steroids	3.17	3.79	3.96 × 10^–6^
2-Piperidinone	Nitrogen compounds	2.51	3.37	1.45 × 10^–5^
Benzamide	Aromatic compounds	2.48	3.80	5.85 × 10^–5^
Isovalerylglycine	Amino acids	2.18	3.33	2.75 × 10^–4^
N-Acetylvanilalanine	Amino acids	2.07	2.64	4.37 × 10^–4^
N-Ethylglycine	Amino acids	2.06	−1.71	5.66 × 10^–3^
Cotinine N-oxide	Nicotine	2.02	−1.59	2.13 × 10^–6^
LysoPC(16:0)	Lysophospholipids	2.00	−1.38	4.24 × 10^–8^
Farnesol	Terpenoids	1.90	−1.32	3.87 × 10^–2^
P1,P4-Bis(5'-uridyl) tetraphosphate	Nucleotides	1.87	−1.28	3.74 × 10^–4^
Citric acid	TCA cycle	1.86	1.62	1.19 × 10^–4^
2,4-Di-tert-butylphenol	Phenols	1.76	−1.06	2.61 × 10^–2^
N-Acetylhistidine	Amino acids	1.69	1.67	3.96 × 10^–6^
5-Dehydroavenasterol	Sterols	1.61	−0.95	5.89 × 10^–3^
Alanyl-Histidine	Dipeptides	1.60	1.76	3.15 × 10^–3^
Chicoric acid	Phenols	1.52	1.73	5.55 × 10^–3^
L-Lysine	Amino acids	1.49	−0.87	9.93 × 10^–4^
Acetamidopropanal	Aldehydes	1.47	1.28	1.45 × 10^–4^
Formylanthranilic acid	Benzoic acids	1.42	−0.83	5.29 × 10^–4^
Gamma-Glutamyl-Se-methylselenocysteine	Amino acids	1.41	−0.68	9.32 × 10^–3^

### Breed-specific blood biomarkers in response to HS

Comparison between the EXP and POST phases in Angus cattle using OPLS-DA (additional file; sheet No.5) revealed a clear multivariate separation between sampling periods, with 264 metabolites contributing to class discrimination (VIP > 1). Among these, urobilin (linked to porphyrin metabolism) emerged as the most influential compound (VIP = 4.311; ROC AUC = 1). Other metabolites contributing to multivariate discrimination included small peptides (e.g., serylarginine, glutamylhistidine, alanylarginine), nucleotide-related compounds, carnitine derivatives, and glycerophospholipids. However, when longitudinal variation was evaluated using linear mixed models accounting for repeated measures and multiple testing correction, only three metabolites remained statistically significant (FDR-adjusted *p* < 0.05), namely urobilin (adjusted *p*-value: 2.81 × 10^–8^), deoxycitidine (adjusted *p*-value: 2.92 × 10^–2^), and D-1-piperideine-2-carboxylic acid (adjusted *p*-value: 2.92 × 10^–2^). Deoxycitidine displayed higher levels during EXP (Log_2_FC = 3.63), while urobilin and D-1-piperideine-2-carboxylic acid showed reduced levels in EXP, recording Log_2_FC values of −6.08 and −3.55.

In Nellore cattle, the EXP vs POST comparison produced a markedly different picture. OPLS-DA identified 277 discriminant metabolites (VIP > 1), showing a strong multivariate separation (additional file; sheet No.6). Importantly, linear mixed model analysis with FDR correction revealed 114 metabolites exhibiting statistically significant phase-dependent variation, indicating extensive metabolic remodeling (additional file; sheet No6.). The metabolites affected in Nellore (Table 2) could be included in several major biochemical classes, such as amino acids and peptides, carbohydrates, purine and pyrimidine derivatives, acyl-carnitines, and steroid-related metabolites.

**Table 2:** Chemical class distribution of FDR-significant metabolites identified in Nellore cattle across experimental phases (EXP vs POST). The table summarizes the number of statistically significant metabolites within each chemical class, the cumulative Log_2_ fold-change (Log_2_FC) describing the overall direction and magnitude of variation between EXP and POST, and the most discriminant metabolite within each class based on OPLS-DA variable importance (VIP score). Only metabolites remaining significant after false discovery rate (FDR) correction (Benjamini-Hochberg) in linear models with covariate adjustment were included

Chemical class	Total compounds	Log_2_FC cumulative (EXP vs POST)	Most discriminant VIP metabolite
Acyl carnitines	4	9.94	Arachidyl carnitine (*p*-value: 9.03 × 10^–3^; VIP score: 1.65; AUC: 0.84)
Amino acids, peptides, and analogues	34	−42.08	Deoxyhypusine (*p*-value: 1.13 × 10^–4^; VIP score: 3.15; AUC: 1)
Benzenoids	2	−5.31	Benzaldehyde (*p*-value: 3.60 × 10^–3^; VIP score: 1.87; AUC: 1)
Carbohydrates and carbohydrate conjugates	13	−23.29	N-Acetyl-glucosamine 1-phosphate (*p*-value: 1.32 × 10^–3^; VIP score: 2.99; AUC: 0.92)
Fatty acyls	7	1.42	Resolvin D1 (*p*-value: 6.07 × 10^–3^; VIP score: 1.82; AUC: 1)
Glycerophospholipids	3	−2.48	PA(16:0/18:1(9Z)) (*p*-value: 2.63 × 10^–2^; VIP score: 1.99; AUC: 0.76)
Pteridines and derivatives	5	−7.79	Tetrahydroxypterin derivative (*p*-value: 4.06 × 10^–3^; VIP score: 2.12; AUC: 0.92)
Purine and pyrimidines	8	−8.73	Citicoline (*p*-value: 1.01 × 10^–3^; VIP score: 3.21; AUC: 0.92)
Pyridines and derivatives	4	−5.85	D-1-Piperideine-2-carboxylic acid (*p*-value: 2.56 × 10^–3^; VIP score: 2.06; AUC: 1)
Steroids and steroid derivatives	7	−10.17	3a,7a,12a-Trihydroxy-5b-cholestanoic acid (*p*-value: 9.94 × 10^–6^; VIP score: 3.60; AUC: 1)
Tetrapyrroles and derivatives	4	−3.48	Urobilin (*p*-value: 2.09 × 10^–6^; VIP score: 3.63; AUC: 1)

**Fig. 2:**
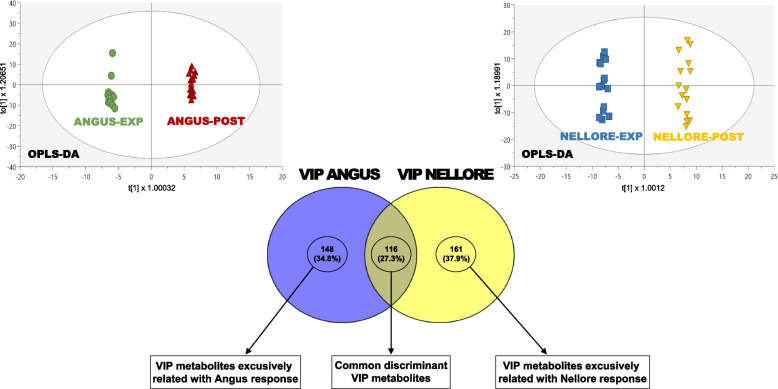
Venn diagram showing the number of exclusive and common blood metabolites affected by HS (EXP vs POST). The discriminating metabolites were extrapolated from two supervised OPLS-DA models built considering both Angus and Nellore breeds

Urobilin displayed significantly lower abundance during EXP compared to POST (Log_2_FC = −5.97; adjusted *p*-value = 2.09 × 10⁻⁶). Amino acid and peptide-related metabolites represented one of the most numerically represented categories among the significant features (34 compounds). Carbohydrate-related metabolites also showed phase-dependent variation. Nucleotide derivatives belonging to purine and pyrimidine metabolism were predominantly reduced during EXP. Steroids and steroid-conjugated metabolites exhibited consistent differences between phases. Phenolic compounds, including quercetin and enterolactone, were also reduced during EXP. Additional metabolites associated with central carbon metabolism and redox-related processes (e.g., phosphoenolpyruvic acid and glutathione-related compounds) showed statistically significant variation across phases (additional file; sheet No. 6). The Venn diagram (Fig. 2) indicated that a subset of VIP metabolites was shared between breeds, although differences in both magnitude and direction of change were observed (additional file; sheet No. 7). Notably, the number of FDR-significant metabolites differed markedly between breeds, with Nellore exhibiting a substantially larger set of statistically robust features than Angus.

### Breed-specific pathway alterations in response to HS

**Fig. 3:**
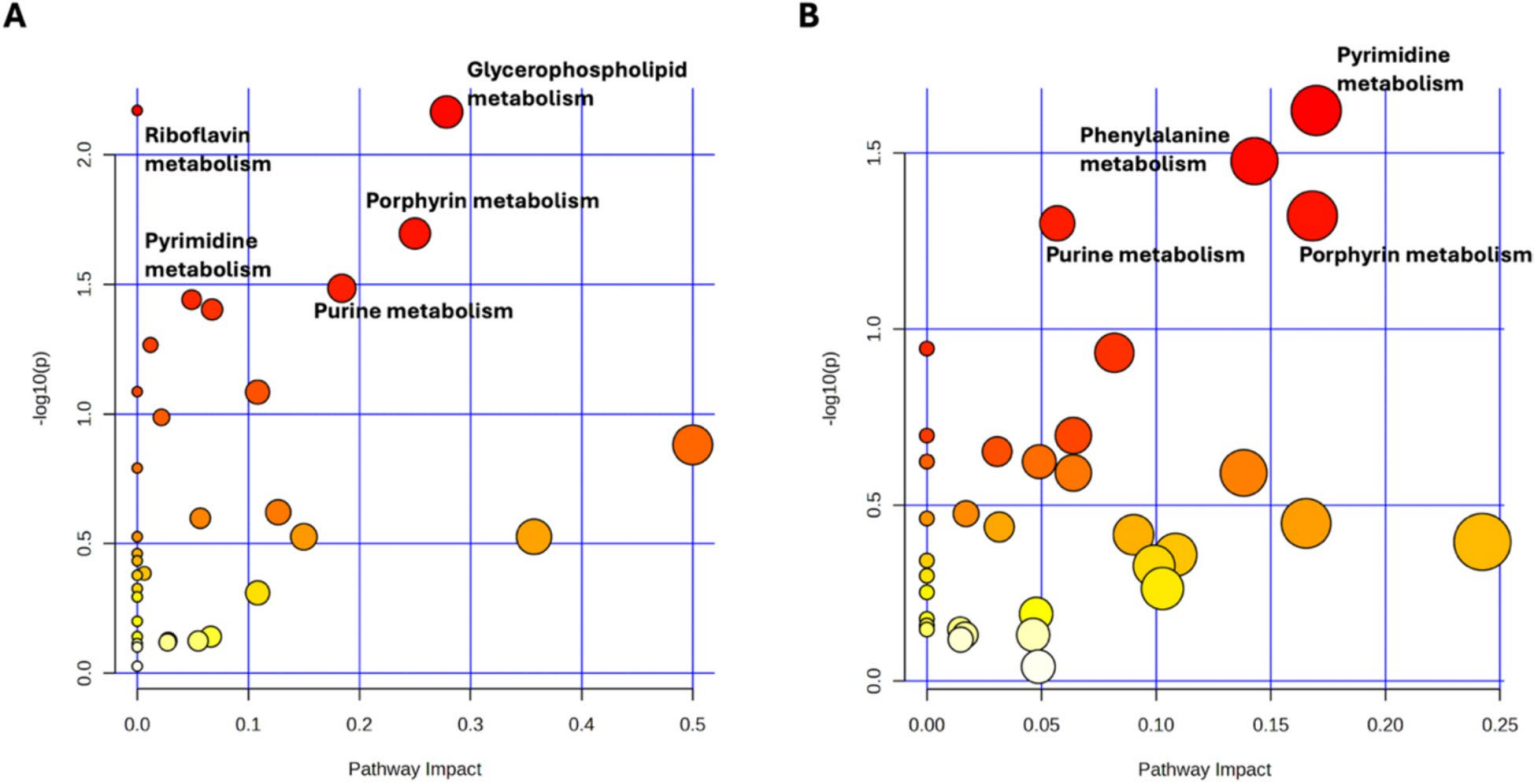
**A** Explorative pathway analysis (against the *B. taurus* metabolome) for the Angus EXP vs POST comparison using the VIP metabolites from OPLS-DA. **B** Explorative pathway analysis (against the *B. indicus* metabolome) for the Nellore EXP vs POST comparison using the VIP metabolites from OPLS-DA

Pathway analyses were initially performed using all metabolites contributing to multivariate discrimination from OPLS-DA (VIP > 1) for each breed, in line with common practices in untargeted metabolomics aimed at capturing coordinated metabolic shifts. This approach highlighted several enriched metabolic routes in both breeds, including porphyrin metabolism, purine metabolism, and pyrimidine metabolism, suggesting that HS and seasonal transition influenced core biochemical functions (Fig. 3). In Angus, glycerophospholipid and riboflavin metabolic pathways were exclusively enriched, while Nellore showed an exclusive enrichment of phenylalanine-related metabolites.

Pathway analysis was subsequently refined by prioritizing metabolites that remained statistically significant after linear mixed model analysis with FDR correction. This filtering step reduced the dataset used for pathway-level evaluation. Under this criterion, Nellore samples retained a larger number of significant metabolites mapping to multiple metabolic pathways, including porphyrin metabolism, purine and pyrimidine metabolism, amino acid-related pathways, and lipid-associated pathways (fatty acyls and glycerophospholipids). In Angus, the number of metabolites surviving FDR correction was markedly smaller (a total of 3 compounds), resulting in fewer pathways meeting statistical relevance criteria. Under these experimental conditions, data acquisition was performed exclusively in positive ionization mode (ESI +); therefore, metabolites preferentially detected under negative ionization conditions, including several classes of organic acids, may be underrepresented. Pathway-level results thus reflect the detectable metabolome under the applied analytical conditions.

## Discussion

In this work, untargeted metabolomics was employed to characterize both breed-dependent and phase-dependent metabolic responses to HS in Nellore and Angus cattle. The inter-breed comparison conducted during the challenge period (EXP), based on independent groups, revealed clear metabolic divergence between breeds, consistent with previous evidence linking HS adaptation to alterations in amino acid metabolism, lipid remodeling, and energy-related pathways (Tian et al. 2015; Jorge-Smeding et al. 2024). These findings align with our earlier observations on blood DNA methylation patterns obtained from the same animals (Del Corvo et al. 2021), supporting the concept that genetic background strongly influences systemic responses to thermal stress. Among the metabolites discriminating breeds during EXP, 4-Hydroxyestrone exhibited the highest prediction ability. Steroid metabolism is known to be highly responsive to environmental stressors (Koch et al. 2016), and differences in this metabolite suggest breed-specific endocrine or steroidogenic regulation under HS conditions. Additional highly predictive biomarkers differentiating Angus and Nellore (Table 1) included lysine, LysoPC(16:0), and citric acid, metabolites previously associated with HS-related metabolic adjustments (Tian et al. 2015; Jorge-Smeding et al. 2024). The observed variations are coherent with the known physiological impact of HS on mitochondrial metabolism, substrate utilization, and membrane lipid turnover (Feng et al. 2022).

While inter-breed comparisons describe inherent metabolic differences, the longitudinal analysis (EXP vs POST) provides mechanistic insight into adaptive dynamics. Here, a striking contrast emerged between breeds. In this regard, Nellore exhibited a broad set of FDR-significant metabolites (i.e., a total of 114 blood metabolites) following linear mixed model analysis with covariate adjustment (Table 2), indicating extensive metabolic reprogramming associated with HS exposure and subsequent recovery. In contrast, Angus displayed only 3 metabolites surviving multiple-testing correction, highlighting a markedly reduced statistically robust metabolic shift across phases. This divergence is biologically meaningful. A larger FDR-regulated metabolite set in Nellore suggests a coordinated and tightly regulated metabolic adaptation, whereas the minimal FDR response in Angus may indicate either higher variability or reduced systemic plasticity. Importantly, absence of numerous FDR-significant metabolites does not imply lack of HS effects in Angus, but rather reflects differences in response structure and robustness.

Class-level interpretation of Nellore responses revealed that HS predominantly affected amino acids, peptides, and analogues, which represented the most impacted chemical family both in compound count and cumulative fold-change magnitude. The global reduction observed for this class is consistent with HS-induced shifts in nitrogen metabolism and amino acid utilization (Jorge-Smeding et al. 2024). HS is frequently associated with altered protein turnover, enhanced amino acid catabolism, and redistribution of substrates toward energy production or stress-related pathways. The predominance of this class underscores the central role of protein and nitrogen metabolism in Nellore’s adaptive physiology. Other metabolite families exhibited coherent patterns. For example, carbohydrates and carbohydrate conjugates showed an overall reduction, suggesting modulation of central carbon metabolism and energy allocation. HS alters glucose utilization, gluconeogenic activity, and glycolytic fluxes (Feng et al. 2022; Koch et al. 2016), making variations in sugar-related metabolites physiologically plausible. Similarly, decreases in purine and pyrimidine derivatives (i.e., metabolites previously reported in literature mainly as related to nitrogen efficiency of cattle (Stentoft et al. 2014; Giagnoni et al. 2025)) support the involvement of nucleotide metabolism during HS adaptation. These pathways are tightly linked to energy balance, redox homeostasis, and stress signaling (Baumgard and Rhoads 2013), and their regulation may reflect shifts in biosynthetic priorities under thermal load. Lipid-related classes displayed distinct behavior (Table 2); particularly, acyl-carnitines showed a strong cumulative increase in Nellore, suggesting altered fatty acid transport and mitochondrial β-oxidation dynamics (Jorge-Smeding et al. 2024). Acyl-carnitines are key intermediates in lipid catabolism, and their accumulation may reflect increased reliance on lipid substrates or transient adjustments in mitochondrial fluxes (Veld et al. 2009). In contrast, several steroids and steroid derivatives were globally reduced, reinforcing the sensitivity of endocrine-related metabolism to HS. These lipid-related metabolic adjustments are particularly relevant when interpreted alongside previous molecular findings obtained in the same experimental model. Particularly, our untargeted metabolomics observations on lipid and membrane-associated metabolites are consistent with earlier evidence reported by Del Corvo et al. (2021), where Nellore cattle exhibited hypo-methylation of stress-responsive genes during HS. Notably, genes involved in fatty acid metabolism and membrane dynamics, including ELOVL5 (Elongation of Very Long Chain Fatty Acids Protein 5) and FADS1 (Fatty Acid Desaturase 1), together with PDE5A, a regulator of vascular function and oxidative stress responses, were differentially methylated. The convergence between epigenetic regulation and the metabolic remodeling observed here supports the hypothesis that Nellore cattle possess a more coordinated adaptive framework to thermal stress. Such integration between gene regulation and metabolite-level responses may contribute to preserving membrane stability, optimizing lipid turnover, and limiting oxidative damage, ultimately reinforcing HS tolerance. This multi-layered perspective offers promising directions for future investigations into the biological basis of heat resilience in livestock.

In Angus, despite the large number of VIP metabolites contributing to class discrimination, the limited number of FDR-significant compounds indicates that metabolic differences were relatively modest at the univariate level, pointing to a weaker or less coordinated systemic metabolic reconfiguration. However, the identity of FDR-significant metabolites provides valuable mechanistic clues. Urobilin exhibited a strong decrease (Log_2_FC = −6.08), with maximal prediction ability (ROC = 1; VIP = 4.311). Urobilin is a downstream product of heme and porphyrin metabolism (Bonkovsky et al. 2013), and its variation suggests that even in the absence of widespread statistical significance, HS influences redox- and heme-related processes (a common metabolic route with Nellore). Alterations in porphyrin turnover are consistent with oxidative and metabolic stress (Ayemele et al. 2021; Zeng et al. 2023). Additionally, the increase of deoxycitidine indicates modulation of nucleoside metabolism, potentially reflecting nucleotide turnover or DNA/RNA-related processes under HS. This latter condition can elevate cellular damage and repair activity, then influencing pyrimidine metabolism. Meanwhile, the reduction of D-1-Piperideine-2-carboxylic acid, a lysine degradation intermediate, suggests perturbations in amino acid catabolic pathways. Although numerically limited, these metabolites collectively point toward HS-induced effects on oxidative metabolism, nucleotide balance, and amino acid turnover.

Pathway-level interpretation should be considered together with the statistical structure of the dataset. In Nellore, the large number of metabolites remaining significant after FDR correction enables more stable inference at the pathway level, suggesting that HS elicits coordinated biochemical adjustments rather than isolated metabolite fluctuations. In Angus, the smaller set of statistically robust metabolites inherently limits pathway over-representation, reflecting the increased stringency of multiple-testing correction rather than inconsistencies across analyses. Consequently, pathway-related observations in Angus are best interpreted as biologically suggestive trends. Despite these differences, porphyrin metabolism consistently emerged as a biologically coherent pathway. This metabolic route is central to heme biosynthesis, redox regulation, and cellular oxidative balance (Bonkovsky et al. 2013), processes intrinsically connected with HS physiology. Thermal stress is known to influence mitochondrial activity (Scerri et al. 2023), oxygen utilization, and reactive oxygen species generation (Ayemele et al. 2021; Zeng et al. 2023), thereby making perturbations of porphyrin-derived metabolites (Obi et al. 2022) mechanistically plausible. The marked variation of heme-related catabolites, including the significant decrease of urobilin, further supports modulation of heme turnover dynamics under HS conditions. The involvement of purine and pyrimidine metabolism is consistent with established stress biology (Ouellet et al. 2021). These pathways play fundamental roles in nucleotide biosynthesis, energy transfer, and cellular signaling (Baumgard and Rhoads 2013), all of which are sensitive to metabolic reprogramming during environmental stress. Notably, the significant variation of purine biosynthesis intermediates such as 5-Aminoimidazole ribonucleotide (AIR) (Caspi et al. 2014), together with nucleoside derivatives observed in Nellore (Table 2), likely reflects adaptive redistribution of nucleotide resources required for stress response, repair processes, and metabolic regulation. Such adjustments are consistent with a systemic metabolic response aimed at preserving cellular homeostasis during heat challenge (Slimen et al. 2015). These observations suggest that Nellore and Angus differ not only in individual metabolite variations but in the overall architecture of their metabolic responses to HS. Nellore displays a broad and statistically robust metabolic reorganization, indicative of coordinated biochemical adjustments and a greater metabolic plasticity under thermal challenge (Nonato et al. 2023; do Nascimento Barreto et al. 2024). In contrast, Angus exhibits a more restricted set of FDR-significant changes, which may reflect alternative regulatory dynamics or a higher degree of biological variability within the breed. Importantly, these findings support the notion that HS tolerance may be more closely associated with the capacity for controlled and systemic metabolic reprogramming rather than with the magnitude of isolated metabolite fluctuations.

## Conclusion

Untargeted UHPLC-HRMS metabolomics enabled a comprehensive characterization of the blood metabolome of Nellore and Angus cattle and revealed distinct metabolic signatures associated with HS exposure and recovery. The integration of multivariate modelling and statistical approaches accounting for repeated measures highlighted that breed-related differences extend beyond individual metabolites and involve broader response-level organization. Nellore cattle exhibited a wide and statistically robust metabolic reconfiguration during HS, consistent with coordinated biochemical adjustments across multiple metabolite classes, including amino acids, nucleotide-related compounds, lipid-derived metabolites, and tetrapyrrole derivatives. In contrast, Angus displayed a more restricted set of FDR-significant variations, suggesting that HS responses may differ in their systemic structure rather than solely in the magnitude of metabolite changes. Pathway-level interpretation indicated biologically coherent modulation of porphyrin, purine, and pyrimidine metabolism, metabolic routes intrinsically linked to redox balance, cellular regulation, and stress physiology. These findings support the view that HS adaptation is associated with controlled metabolic reprogramming and network-level regulation rather than isolated metabolite fluctuations. Collectively, this study provides mechanistic insights into breed-specific metabolic dynamics under thermal challenge and identifies candidate metabolites and biochemical classes potentially relevant to thermotolerance biology. Given the exploratory nature of the study and the limited sample size, future investigations based on larger cohorts and targeted validation strategies will be essential to confirm the biological roles of the identified metabolites. Expanding integrative multi-omics frameworks will further contribute to elucidating the complex regulatory mechanisms underlying HS resilience in livestock.

## Materials and methods

### Experimental design and data collection

The study involved 25 Nellore (*B. indicus*) and 25 Angus (*B. taurus*) healthy young males, approximately 15 month-old, bullocks. Half-sib individuals within each breed were selected to minimize genetic variation. Particularly, Angus and Nellore bullocks were purchased in different regions of Brazil and transported to the experimental station of the Veterinary Faculty, Universidade Estadual Paulista – UNESP, Araçatuba, Brazil at 7 to 10 months of age (Del Corvo et al. 2021). A comprehensive description of the diet fed during this study is given in our previously published work (Del Corvo et al. 2021). Briefly, from arrival (i.e., June 13, 2015, for Angus and September 2, 2015, for Nellore) until October 2, 2015, animals were reared on *Brachiaria brizantha* pasture, with shadow available and water access. Gradually they were adapted to semi-confined and to a diet based on citrus pulp, ground corn, soybean meal and urea, as well as mineral supplementation, in the proportion of 2.5% of live weight. Finally, they were confined to four lots with access to shade and received a same diet based on sugarcane bagasse 45 (Êxito Rural®, with a ratio forage: concentrate 55:45) administered twice a day and water at libitum. The experimental protocol had three phases:

#### Adaptation period (PRE)

Animals were randomly assigned to two groups per breed and housed in 200 m^2^ paddocks, with 100 m^2^ covered by an 80% sunblock shade net, from October 3 to December 3, 2015 (60 days). They had regular access to pasture.

#### Challenge period (EXP)

On December 4, 2015, the shade net was removed from one Angus and one Nellore paddock, and half of the animals were exposed to direct sunlight and high temperature-humidity conditions until February 3, 2016 (56 days).

#### Recovery period (POST)

On February 4, 2016, shading was restored, and animals were kept with shade and pasture access until they were slaughtered in June 2016.

As far as data collection is concerned, environmental data were collected hourly from 01/06/15 to 31/10/16 from CETESB meteorological station located at the Veterinary Medicine campus of the San Paolo State University (UNESP) Araçatuba, nearby the experimental site. Experimental variables collected were Temperature, (T in °C), Humidity (H as % values), solar radiation (R in W/m^2^) and wind speed (W in m/s). As reported in our previous work (Del Corvo et al. 2021), from environmental data, we calculated THI (Temperature Humidity Index) which considers Temperature and Humidity in a single index (Del Corvo et al. 2021), revealing that Angus and Nellore breeds exposed to direct sunlight and high temperature-humidity conditions were under HS (EXP).

### Collection and analyses of blood samples

As reported by Del Corvo et al. (2021), Nellore cattle completed their acclimation by November 2015, during a period of hot and humid weather that induced moderate thermal stress. Blood samples were collected via jugular venipuncture from five sunlight-exposed animals per breed (Nellore and Angus) in early February 2016, corresponding to peak heat stress conditions (EXP period), and again in mid-June 2016 (POST period), during the cooler season after recovery. As explicitly stated in Del Corvo et al. (2021), animals were randomly selected within each breed from the sun-exposed group. Animals were not fasted prior to sampling; the total mixed ration was administered in the morning, and blood collection was performed in the early afternoon. Sampling time was kept consistent across experimental phases to minimize potential circadian effects. Blood was collected into sodium-EDTA tubes as anticoagulant, immediately frozen in liquid nitrogen, and stored at − 80 °C until metabolomic analysis. In this study, whole blood was analyzed to capture both plasma and intracellular metabolites, enabling a comprehensive assessment of systemic metabolic responses to heat stress. The blood metabolomic profiles of the same 10 animals previously characterized for DNA methylation patterns were evaluated (Del Corvo et al. (2021). All experimental procedures complied with ethical guidelines for animal research and were approved by the Ethics Committee on the Use of Animals (CEUA) of the Universidade Estadual Paulista (FOA; approval number 2014–01445). The study is reported in accordance with ARRIVE guidelines (https://arriveguidelines.org).

### Extraction of blood metabolites

Metabolomic samples were prepared as described previously by Teruya et al. (2021), with some modifications. Briefly, the blood samples were thawed at room temperature, and then a 0.2 mL aliquot was extracted in 1.8 mL of 80% methanol using ultrasound-assisted extraction (15 min, 120 Watt, room temperature). After mixing, samples were transferred to Amicon Ultra 3-kDa cutoff filters (Millipore) and centrifuged (30 min, 4100 × g) to remove proteins and cellular debris. After sample concentration by vacuum evaporation (SpeedVac), each sample was resuspended in 50 µL of 50% acetonitrile, and 2 µL were used for injection into the UHPLC-HRMS system.

### Untargeted metabolomics based on UHPLC-HRMS analysis

The untargeted UHPLC-HRMS analysis was done using a Q Exactive Focus Hybrid Quadrupole-Orbitrap Mass Spectrometer (Thermo Scientific, Waltham, MA, USA) coupled to a Vanquish ultra-HPLC pump, equipped with heated electrospray ionization (HESI)-II probe (Thermo Scientific, USA). A detailed description of the instrument settings can be found in Cattaneo et al. (2023). Overall, the chromatographic separation was achieved using an Ultra-Performance Liquid Chromatography column functionalized with Octadecyl Silane using the Bridged Ethylene Hybrid technology (ACQUITY UPLC Waters BEH C18; 2.1 × 100 mm, 1.7 µm), with a 6–94% acetonitrile elution gradient in 35 min and 0.1% formic acid as phase modifier. The flow rate was 200 μL/min, injecting 2 μL of each sample. The untargeted analysis was done in full scan, in the range 100–1200, using a positive ionization mode and setting a nominal mass resolution of 70,000 FWHM (Full Width Half Maximum). Additionally, pooled quality control (QC) samples were randomly injected and analyzed in data-dependent MS/MS (Tandem Mass spectrometry) mode (Top *N* = 3, under a stepped normalized collisional energy) with a full-scan mass resolution of 17,500 FWHM. The HESI (Heated Electrospray Ionization) parameters, together with automatic gain control values and maximum injection times, were as described by Rocchetti et al. (2022). The raw data were further processed using the software MS-DIAL (version 4.90) for automatic peak finding, LOWESS (Locally Weighted Scatterplot Smoothing) normalization, and annotation via spectral matching against the comprehensive Bovine Metabolome Database (Rocchetti et al. 2022; Foroutan et al. 2020) (last accessed date: July 2024). The identification step was based on mass accuracy, isotopic pattern, and spectral matching, reaching annotation confidence levels 2 and 3 (García-Pérez et al. 2024). The raw abundance values of each annotated metabolite were complemented by additional information, including annotation confidence level, reference and measured m/z, total identification score, InChIKey (International Chemical Identifier Key) specifications, MS1 (Precursor Ion Scan) isotopic profile, and MS/MS spectra. The reproducibility was evaluated by calculating the Relative Standard Deviation (RSD %) of each annotated metabolite in the randomly injected QC sample (Ghosh et al. 2021). The metabolomics data have been deposited to MetaboLights (Yurekten et al. 2024) repository with the study identifier MTBLS12545.

### Multivariate statistical analyses

The raw UHPLC-HRMS (Ultra-High-Performance Liquid Chromatography coupled with High-Resolution Mass Spectrometry) data were evaluated using MetaboAnalyst 6.0 (Pang et al. 2024) for unsupervised and supervised statistical analyses. As a first step, the relative abundance values of each annotated blood metabolite were normalized by median, Log_10_ transformed, and Pareto scaled. The unsupervised statistics were based on hierarchical cluster analysis (HCA; distance measure: Euclidean; clustering method: Ward) and principal component analysis (PCA). The supervised statistics were based on orthogonal projections to latent structures discriminant analysis (OPLS-DA), considering different prediction models for each comparison. The OPLS-DA model parameters (namely goodness-of-fit R^2^ and goodness-of-prediction Q^2^) were also inspected, considering as a minimum significant threshold for prediction a Q^2^ value > 0.5. Thereafter, each OPLS-DA model was excluded for significant and suspect outliers using a Hotelling's T^2^ test;* p* < 0.05, while a permutation test (*N* = 100) was used to evaluate model over-fitting. To identify the most discriminating metabolites for each comparison, we used variable importance in projection (VIP) method, setting as a minimum significant threshold a VIP score > 1. Volcano plots were generated using Fold-Change (FC) thresholds (cut-off value > 1.2) combined with analysis of variance (ANOVA)-derived *p*-values (*p* < 0.05). To evaluate candidate metabolites related to HS, Receiver Operating Characteristics (ROC) curves were created using the software MetaboAnalyst 6.0 (Yurekten et al. 2024). The area under the ROC curve (AUC) was inspected to evaluate the global performance of each VIP marker. Following the recommendations of Xia et al. (2013), the utility of each biomarker for discrimination purposes based on its AUC value was considered as follows: 1.0–0.9 = excellent; 0.8–0.9 = good; 0.8–0.7 = fair; 0.7–0.6 = poor; 0.6–0.05 = fail. Repeated measurements were handled using linear models with covariate adjustment implemented in MetaboAnalyst 6.0. Time was treated as the main explanatory variable, and Subject (animal ID) was included as a covariate to account for subject-specific effects arising from longitudinal sampling. By conditioning the model on Subject, the analysis controls for baseline metabolic differences between animals and reduces confounding due to intra-individual correlations. *P*-values obtained from linear models were adjusted for multiple testing using false discovery rate (FDR) correction according to the Benjamini–Hochberg procedure.

### Pathway analyses

The online tool MetaboAnalyst 6.0 was used to inspect the most represented pathways from the discriminating annotated metabolites (using as pathway library: *B. taurus* and *B. indicus*, Kyoto Encyclopedia of Genes and Genomes, KEGG) and provide a metabolite set enrichment analysis based on both discriminating metabolites and metabolic pathways, as identified by the multivariate statistical approach used.

## Supplementary Information

Supplementary Material 1. The supplementary material is given in an excel file with the following sheets: 1) Blood metabolites annotated using the untargeted UHPLC-HRMS approach; 2) Unsupervised clustering and dendrogram built considering all the annotated metabolites; 3) Supervised OPLS-DA score plot built considering all the annotated metabolites; 4) Supervised OPLS-DA score plot built considering only the challenge period; 5) Discriminant VIP metabolites from the EXP *vs *POST comparison for Angus breed, considering also the results from linear models with covariate adjustment; 6) Discriminant VIP metabolites resulting from the EXP *vs* POST comparison for Nellore breed, considering also the results from linear models with covariate adjustment; 7) Venn diagram showing VIP metabolites found in both breeds that were affected by HS.

## Data Availability

Raw data are available via MetaboLights with identifier MTBLS12545. The Dataset is also provided within the supplementary information files.

## Abbreviations

AIR: 5-Aminoimidazole ribotide
ANOVA: Analysis of variance
AUC: Area under the curve
BMDB: Bovine Metabolome Database
CEUA: Ethics Committee on the Use of Animals
Cum: Cumulative value
DNA: Deoxyribonucleic acid
ELOVL5: Elongation of Very Long Chain Fatty Acids Protein 5
EXP: Challenge period
FADS1: Fatty Acid Desaturase 1
FC: Fold Change
FWHM: Full width half maximum
HCA: Hierarchical cluster analysis
HESI: Heated electrospray ionization
HRMS: High-resolution mass spectrometry
HS: Heat stress
InChIKey: International Chemical Identifier Key
KEGG: Kyoto Encyclopedia of Genes and Genomes
Log_2_FC: Binary logarithm of Fold Change
MS: Mass spectrometry
MS-DIAL: Mass Spectrometry Data Independent AnaLysis
MS/MS: Tandem Mass Spectrometry
MS1: First stage of mass spectrometry (precursor ion scan)
MS2: Second stage of mass spectrometry (fragment ion scan)
OPLS-DA: Orthogonal projections to latent structures discriminant analysis
PCA: Principal Component Analysis
PDE: Phosphodiesterase
PDE5A: Phosphodiesterase 5A
PLS-DA: Partial least square discriminant analysis
POST: Recovery period
PRE: Adaptation period
Q^2^: Predictive Ability Score in OPLS-DA Models
R^2^: Goodness-of-Fit Score in Statistical Models
RNA: Ribonucleic acid
ROC: Receiver-operating characteristic
T^2^: Hotelling's T-Squared Test (Multivariate Statistical Test)
TCA: Tricarboxylic acid cycle
UHPLC: Ultra-high-performance liquid chromatography
UHPLC-HRMS: Ultra-High-Performance Liquid Chromatography coupled with High-Resolution Mass Spectrometry
VIP: Variable importance in projection
